# Differential impact of trait, social, and attachment anxiety on the stare-in-the-crowd effect

**DOI:** 10.1038/s41598-019-39342-8

**Published:** 2019-02-11

**Authors:** Nicolas Burra, Solene Massait, Pascal Vrtička

**Affiliations:** 1 Faculté de Psychologie et des Sciences de l′Education, Université de Genève, Geneva, Switzerland; 2 Max Planck Institute for Human Cognitive and Brain Sciences, Research Group Social Stress and Family Health, Leipzig, Germany

## Abstract

Eye gaze conveys crucial information for social interactions, with straight versus averted gaze triggering distinct emotional and cognitive processes. The “stare-in-the-crowd” effect exemplifies such differential visual processing of gaze direction, in more recent reports also in interaction with head orientation. Besides aiming at replicating the “stare-in-the-crowd” effect by means of an eye gaze by head orientation interaction, the present study intended to for the first time testing its susceptibility to inter-individual differences in trait, social, and attachment anxiety. Our findings reveal a significant relation between the “stare-in-the-crowd” effect and social and attachment, but not trait anxiety, and therefore provide preliminary cues for personality influences on visual processing of eye gaze and head orientation.

## Introduction

When initiating social interactions, humans rely upon information from other people’s faces and in particular their eyes. We decide when and how to interact with others by instigating eye contact with them^1^ or following their eye gaze^2,3^ because the eyes of others communicate subtle but crucial signals about their intentions and goals^4–6^. Not surprisingly, the very first fixation usually lands on the eye region of a facial image. Furthermore, “internal” features of the face – and particularly eye gaze – are looked at more than “external” features such as hair, forehead, or ears^7–9^. Eye perception thus is overemphasized as compared to other facial features during social interaction.

From an evolutionary perspective, it is argued that the processing of eye gaze has likely developed as a mechanism of (predatory) threat detection, because “… awareness of the direction in which another person is attending provides critical information … for assessing potential sources of threat”^10^. By attending to the gaze of others, the observer obtains an indication of others’ “… direction of attention and focus of interest in the surrounding space”^11^. Such monitoring of others’ attention is not only important regarding threat detection, but also conveys valuable information for social interaction. Within this context, research on eye gaze has prominently focused on the psychological and neural mechanisms of direct/straight- versus averted-gaze detection, because the latter two gaze directions trigger distinct cognitive processes^11^.

In the case of straight gaze, social psychology emphasizes its function in the synchronization between individuals, as straight gaze signals mutual attention^12^. A straight gaze is therefore usually associated with approach-oriented emotions^13^. Approach-oriented emotions, however, can have various positive and negative meanings, ranging from the expression of intimacy to dominance, social control, or social evaluation, the latter phenomenon referred as to the “watchful-eye” effect^14^. For example, watchful eyes have been found to heighten social awareness and self-evaluation^15,16^, which is in line with the well-established finding that people tend to be on their best behavior when they know that they are being observed by others (see e.g.^17–19^). At the same time, it was found that straight (rather than averted) gaze is often perceived as aggressive and threatening, readily eliciting negative emotions such as anxiety, nervousness, distress, worry, and fear^10,14,20,21^. Panagopopous and Van der Linden^14^ therefore suggest that the watchful-eye effect may ultimately come from a feeling of being watched or socially evaluated, a condition that appears to be predominantly associated with negative subjective emotional states.

What is concerning averted gaze, its function is mainly linked to the induction of an automatic shift of the observer’s spatial attention in the seen gaze direction – that is at the surrounding environment^11^. Yet, an averted gaze has also been found to influence the processing of facial displays with averted gaze *per se*, including judgments about emotions, likeability, or attractiveness, mostly in a negative direction^11^. There is, furthermore, general agreement that an averted gaze is more prominently used to convey avoidance-oriented emotions and/or submissive/fear displays^13^.

By directly comparing the significance of a straight versus averted gaze, von Grünau and Anston^22^ first reported what has been known since then as the “stare-in-the-crowd” effect. In their paradigm, participants were asked to detect either a straight-gaze target amongst averted-gaze distractors or an averted-gaze target amongst straight-gaze distractors. Results revealed that there was an overall faster detection of/response latency advantage for straight-gaze (versus averted-gaze) targets reflecting a search efficiency effect. Furthermore, by manipulating the number of distractor items, a search asymmetry effect emerged because “… the inclusion of additional distractor items had a smaller effect on search latency for the straight-gaze target than it did for the averted-gaze target. In other words, search for the straight-gaze target was more efficient than search for the averted-gaze target”^23^. One or both of the above effects has/have since been replicated^22,24–28^. More recently, an additional factor was added to the “stare-in-the-crowd” paradigm by means of head orientation (frontal versus deviated). In so doing, findings regarding search efficiency revealed that the response latency was longest and the % error rates were highest for the detection of averted- (versus straight-) gaze targets when the eyes and the head create an angle (i.e. deviated head)^24–26^ - but see^29^. This finding was interpreted as reflecting different decoding processes related to the intentional nature of straight versus averted gaze on a frontal versus deviated head^24^. No search asymmetry effect, however, has (yet) been reported with the additional head deviation factor (see^23^). Relying upon the same paradigm as used by Conty *et al*.^24^ including frontal and deviated head orientations, one aim of the present study therefore was to investigate the presence of a search asymmetry effect when including both frontal versus deviated head displays – beyond replication of the previously described head orientation by eye gaze interaction.

Besides testing for a search efficiency and asymmetry effect as such, the present investigation also intended to assess the susceptibility of the “stare-in-the-crowd” effect to inter-individual differences in terms of contextual variables as investigated in prior research^30–35^. In so doing, we were especially interested in anxiety, with anxiety being generally defined as a state of uncertainty about future negative events^36^. Because eye gaze can signal both nonsocial (predatory) threat as well as social threat in terms of being watched or evaluated (see above), we set out to discriminate the influence of three distinct kinds of anxiety on the “stare-in-the-crowd” effect: trait anxiety, social anxiety, and attachment anxiety.

As already pointed out at the beginning of the introduction, it is argued from an evolutionary perspective that the processing of eye gaze has likely developed as a mechanism of (predatory) threat detection^10,11^. If such evolutionary advantage of attending to the eye gaze of others exists, the question is warranted why it would only be beneficial for some but not others. One potential explanation may be provided by *Social Defense Theory* (SDT) suggesting special adaptive advantages of inter-individual differences in threat detection that tend to increase the inclusive fitness of people in groups^37^. SDT relates these inter-individual differences particularly to attachment orientations (see below), but they may also reflect other variance in threat-detection at the individual level. Regardless of the specific psychological construct behind such inter-individual variance, the overall idea is that certain personality characteristics may represent distinct disadvantages that decrease inclusive fitness if they are not complemented by contributions of other people with different – potentially more adaptive – personality traits at the group level.

Within this context, the first type of anxiety we considered was *trait anxiety*. Trait anxiety refers to the stable tendency to attend to, experience, and report negative emotions such as fears, worries, and anxiety across many situations, including both social and nonsocial settings^38^. Prior results revealed a critical role of trait anxiety in the detection of emotional stimuli. In visual search experiments usually containing threatening (snakes and spiders) and non-threatening (mushrooms and flowers) nonsocial images, the search speed for threatening stimuli is shorter^39,40^. This means that search efficiency – as measured by the time spent on each stimulus and known as the visual search slope – is better (i.e. the visual search slope is steeper) for threatening than non-threatening nonsocial information. Such mechanism allows for rapid identification of, as well as the preparation of fast and appropriate responses to, potential threats through attentional capture. Available findings suggest that high trait-anxious individuals show a pattern of responding in which they initially allocate their attention towards a threatening stimulus, but later avoid it^41–43^. In this vein, anxiety-related speeded target detection of threat has been observed in some visual search studies^40,44,45^ – but see^46^. Thus, instead of a facilitated detection of threatening information (i.e. a steep search slope), disengaging from such information might be costly^47^ and lead to the opposite pattern (i.e. a shallow search slope), an effect probably due to stronger attentional control involved in attentional disengagement^44,45^. In terms of an interaction between eye gaze and trait anxiety, one previous study reported an enhanced orienting to the eye gaze of faces with fearful expressions as well as stronger attention capture of angry faces with a straight gaze^48^, and thus an effect of trait anxiety on social information processing. We are, however, not aware of any findings specifically pertaining to trait anxiety and the “stare-in-the-crowd” effect.

The second type of anxiety we assessed was *social anxiety*, which is defined as “fear and avoidance in social and performance situations”^49^ or “an intense fear of evaluation from others in social situations”^50^. Such anxiety of social evaluation manifests itself in its most severe form as social anxiety disorder (SAD)/social phobia. Regarding eye gaze, SAD has been shown to be associated with fear and avoidance of eye-to-eye contact (see e.g.^51–54^, also as a clinical symptom^55^). The latter mechanism is thought to represent a means to preclude the exposure to social evaluation that is perceived as a threat^55–59^. Although we are not aware of any reports on the influence of social anxiety on the “stare-in-the-crowd” effect, one previous study found that participants with higher levels of social anxiety accepted a wider range of gaze deviations from the direct gaze as eye contact^5^.

Finally, the third type of anxiety we considered was *attachment anxiety*. Attachment anxiety is characterized by chronic fear of social rejection or abandonment and a strong desire for close social relationships. It is thought to develop due to repeated unpredictable or inconsistent early child-caregiver interactions so that the child must intensify his/her proximity seeking attempts in order to attain a feeling of security. The latter behavior is associated with a so-called secondary attachment strategy entailing chronic hyperactivation of the attachment system, which is mainly linked to an increased sensitivity to negative social clues that could signal social rejection or abandonment^60^. In line with attachment theory, attachment anxiety in adults has been shown to increase subjective arousal and decrease dominance/controllability ratings of social negative images and video clips^61,62^ and to generally influence attentional mechanisms in terms of a heightened processing of attachment-related information – as measured with different lexical decision and Stroop tasks (see^62^). What is concerning specific relations between attachment and eye gaze processing, surprisingly little literature is available. Although it is generally understood that eye-to-eye contact is an important determinant of infant-caregiver attachment quality through its relation to infant-caregiver synchrony probably mediated by oxytocin^63,64^, we are not aware of any literature pertaining to attachment anxiety and eye gaze processing, including the “stare-in-the-crowd” effect.

To measure the influence of trait, social, and attachment anxiety on eye gaze detection, we employed an adapted version of a visual search task revealing the “stare-in-the-crowd” effect. During this visual search task, participants must identify one deviant target (i.e. averted-gaze) among a number of distractor items (i.e. straight-gaze). In the present experiment, we used stimuli only consisting of the eye region of a face, a procedure that allows for assessing the detection of eye gaze without the interference of other facial features and/or overall expression of the face^22,24,65^. Eye gaze was either straight or averted on a frontal or deviated head^24^. Moreover, we controlled for the distance between stimuli^23,28^ by using an array of items instead of a spread over the entire screen^66^. We measured response times and accuracy (% error rates) across two set sizes (4 and 8) to investigate search efficiency, and response times and accuracy as an interaction with set size to investigate search asymmetry.

According to the above considerations and the original findings of Conty *et al*.^24^ using a very similar experimental setup, we expected to replicate one aspect of the “stare-in-the-crowd” effect across the entire sample of participants. That is, a more efficient detection of a straight-gaze target among faces with an averted gaze (as compared to an averted-gaze target among faces with a straight gaze), but only for eyes with a deviated head orientation – reflected in both response times and % error rates. We were also interested in determining the presence or absence of a search asymmetry effect that has not (yet) been reported when including the additional experimental factor head orientation. Finally, regarding individual differences in trait, social, and attachment anxiety, predictions were difficult, because we were only aware of very few extant studies testing for personality influences of eye gaze detection, particularly in the context of the “stare-in-the-crowd” effect. However, because social and attachment anxiety represent social types of anxiety (in contrast to trait anxiety reflecting a more general tendency to differentially respond to social and nonsocial information) and eye gaze (in combination with head orientation) being thought to convey different kinds of social intent, we expected social and attachment anxiety to more prominently modulate observed effects than trait anxiety.

## Methods

### Participants

52 healthy right-handed students from the University of Geneva were recruited for the present investigation, which was approved by the ethics committee of the Department of Psychology and Educational Sciences at the University of Geneva, in accordance with the Declaration of Helsinki. All participants provided written informed consent and participated in this experiment for class credit. Data from one participant had to be removed due to missing conditions (only half of the experiment was performed), leaving data from 51 participants for data analysis (21 males, age range 18–26, mean = 20.63, SD = 1.43).

### Questionnaires

The present study employed three self-report questionnaires assessing different kinds of trait, social, and attachment anxiety. The questionnaires were as follows. *Trait anxiety* (STAI-T) was assessed using the State-Trait Anxiety Inventory^67^^,^French version^68^. *Social anxiety* was evaluated using the self-report version of the Liebowitz Social Anxiety Scale (LSAS) French self-report version^69^. Finally, *attachment anxiety* (AAX) was assessed using the Relationship Scales Questionnaire (RSQ)^70^, French version^71^ and analyzed according to^72^. Reliability was good for all questionnaires (STAI-T: cronbach’s α = 0.88; LSAS total score: α = 0.94; RSQ AAX: α = 0.69; RSQ AV: α = 0.66).

### Materials and Apparatus

The experiment was run on a 17-inch (1280 by 1024 pixels; 60 Hz refresh rate) LCD monitor. Stimulus presentation and recording of behavioral measures were controlled by Matlab using the Psychophysics Toolbox v.3 extension^73,74^.

The experimental paradigm consisted of a visual search task made up of pictures of the eye region of a face at 6° of the central fixation^24^. Stimuli were taken from George *et al*.^65,75^. The following experimental conditions were manipulated: (1) *set size* – four or eight face stimuli present at a time; (2) *target* (presence versus absence) – stimuli during one trial either consisted of four or eight identical pictures, or contained one deviant picture amongst three or seven remaining identical pictures; (3) *head orientation* – eyes were either superimposed onto a frontal or deviated head^24^; and (4) *eye gaze* – the target could either be a straight gaze surrounded by averted gazes or an averted gaze surrounded by straight gazes^22,24,66^. Averted gaze could be either to the left or right.

All stimuli were matched for mean luminance and contrast using the SHINE toolbox (MATLAB 2013a; Math Works Inc.^76^). Each stimulus measured ~2.9° by 1.6°degrees, which is comparable to previous experiments. Participants were required to respond to the presence or absence of a target using two keyboard buttons with their right hand. Response mapping was counterbalanced across participants.

### Procedure

Participants were seated 54 cm away from the monitor in a dimly lit room. They were informed that the purpose of the experiment was to measure their speed and accuracy in a visual search task consisting of stimuli made up from the eye part of faces. The experiment was split into two parts each containing four random blocks.

Our stimuli were identical to the stimuli used by Conty *et al*.^24^, consisting of the eye region from 20 different individuals (10 female). For each individual face identity, there were four different conditions regarding (i) eye direction (straight towards the camera/observer or averted by 30◦) and (ii) head orientation (frontal or rotated/deviated by 30◦ from the camera/observer). Right and left versions of the stimuli were obtained by mirror imaging.

All combinations were randomized within each block. The block assignment of frontal vs. deviated head orientation and target gaze direction (straight vs. averted) was counterbalanced. Thus, there were eight stimulus configurations for each face identity obtained by crossing head orientation (frontal vs. deviated), gaze direction (averted vs. straight), and side of deviation (left vs. right).

As in Conty *et al*.^24^, two head orientations (half frontal/half deviated) and two target gaze directions (half straight/half averted) were presented in sixteen separate blocks of 40 trials each. The first eight blocks used a frontal head orientation, followed by eight blocks with a deviated head orientation. This assignment was reversed for half the participants. Within each block, the visual search task consisted equally of the display of 4 or 8 stimuli distributed in an array at 6.45° from the center. Within each block, participants were required to detect – as quickly and accurately as possible – the presence (50%) or the absence (50%) of a specific target (straight or averted gaze).

**Figure 1:**
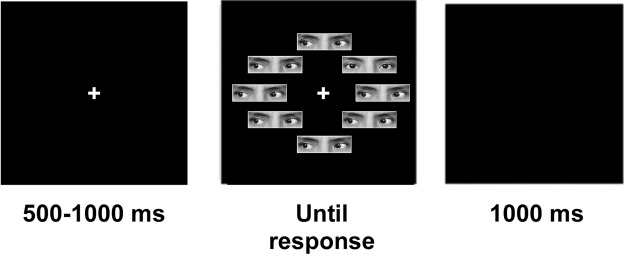
Experimental design. After a jittered fixation cross, a visual search screen was presented until response, followed by a blank screen for 1000 ms. In the present example, the set size is 8 with one frontal head straight-gaze target and 7 frontal head averted-gaze distractors. Please note that eyes of the corresponding author were only used for illustrative purposes, whereas Conty *et al*.^24^ stimuli were actually used in the experiment.

Each trial started with a fixation cross presented for a random duration of 500 to 1000 ms, followed by the visual search task that lasted until the participant provided an answer. In the case of a wrong answer, feedback was provided and displayed in the center of the screen, reading: “mauvaise réponse” (“incorrect response” in French). A black screen was subsequently displayed for 1000 ms preceding the next trial (see Fig. 1).

### Statistical analyses

We first calculated mean scores of questionnaire measures and their interrelations (reported in Table 1 below), as well as mean scores of behavioral measures (response times and accuracy/% error rates) using raw data for both target present and absent conditions (shown in the Supplement: Section S1, Table S1 and Fig. S1). For statistical analyses, we then only focused on the target present conditions and inspected the corresponding response times and % error rates as well as all questionnaire measures for their distribution and presence of outliers. Response times were right skewed for some conditions, so that an ln-transformation was performed. This procedure markedly improved data distribution. We then checked the ln-transformed values for outliers, the latter defined as values above or below three standard deviations from the mean, and winsorized any values meeting such criteria up or down to the respective upper or lower boundaries. All statistical analyses were subsequently computed with the ln-transformed and winsorized response time values. % Error rates were not particularly skewed but contained a few outliers (same as defined above for response times). These were again winsorized up or down to the respective upper or lower boundaries. All statistical analyses were subsequently computed with the winsorized % error rate values. There were no distribution issues with any questionnaire scores, and no outliers were detected, either. Questionnaire scores as well as participant age were centered (z-scored) for statistical analyses.

**Table 1:** Mean scores (with standard deviation [SD]) and correlation between anxiety questionnaire scores.

Questionnaires	Mean (SD)	1	2	3
1. Trait Anxiety (STAI-T)	49.06 (3.68)	—	0.257^(†)^	0.173
2. Social Anxiety (LSAS)	42.80 (19.40)	—	—	0.044
3. Attachment Anxiety (AAX)	2.01 (0.69)	—	—	—

^(†)^*p* < 0.10 (two-tailed). N = 51, Pearson.

**Figure 2:**
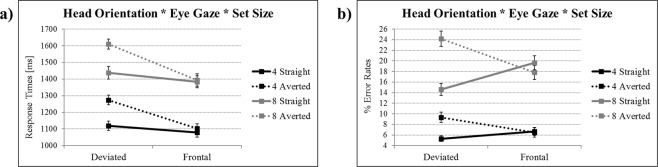
Illustration of the response times and % error rates findings from the 2 × 2 × 2 ANOVA for the target present condition without personality. Panel (a) shows the significant two-way interaction between head orientation by eye gaze (in the absence of a three-way head orientation by eye gaze by set size interaction) for reaction times in milliseconds (back-transformed values), and panel (b) depicts the significant three-way interaction between head orientation by eye gaze by set size for % error rates. Solid lines indicate straight-gaze targets/averted-gaze distractors, and dotted lines indicate averted-gaze targets/straight-gaze distractors. Set size 4 is depicted in black and set size 8 in gray. Error bars represent 1 S.E.M. Depicted are estimated means from the respective 2 × 2 × 2 ANOVA.

Response times and % error rates were first analyzed with a 2 (head orientation) × 2 (set size) × 2 (eye gaze) repeated measures analysis of variance (ANOVA) by controlling for participant age and sex, regardless of individual differences. Search efficiency effects were thereby defined as main effects of head orientation and eye gaze, as well as their interaction, and search asymmetry effects as interactions of the above main effects and interactions with the additional factor set size. In a following step, all questionnaire scores were added as covariates and allowed to simply interact with the experimental factors. For illustration of interactions between experimental factors and questionnaire scores, means were estimated at +/− 1 standard deviation from the mean for the questionnaire score of interest. Because all significant main effects and interactions observed by means of the 2 × 2 × 2 ANOVAs without inclusion of questionnaire scores remained unchanged when calculating the subsequent 2 × 2 × 2 ANOVAs comprising all questionnaire scores, only the results of the latter analysis are reported in Table 2. The results of the 2 × 2 × 2 ANOVAs without inclusion of questionnaire scores are nonetheless illustrated in Fig. 2.

**Table 2:** Results of the two ANOVAs with the factors head orientation, eye gaze, and set size, comprising the three questionnaire scores for (i) trait anxiety (STAI-T), (ii) social anxiety (LSAS), and (iii) attachment anxiety (AAX), regarding response times (top) and % error rates (bottom). All significant interactions are highlighted in bold. The same main effects of, and interactions between eye gaze, head orientation and/or set size were also present for the two ANOVAs not including questionnaire scores.

Factor	F-Value	p-Value
**Response Times**		
**Set Size**	**626.879**	**<0.001**
Set Size * STAI-T	1.299	0.26
Set Size * LSAS	0.78	0.382
Set Size * AAX	0.875	0.354
**Head Orientation**	**42.019**	**<0.001**
Head Orientation * STAI-T	0.05	0.824
Head Orientation * LSAS	2.767	0.103
Head Orientation * AAX	1.762	0.191
**Eye Gaze**	**29.405**	**<0.001**
Eye Gaze * STAI-T	0.5	0.483
Eye Gaze * LSAS	0	0.986
Eye Gaze * AAX	0.094	0.76
Head Orientation * Set Size	0.013	0.909
**Head Orientation** * Set Size * **STAI-T**	**5.993**	**0.018**
Head Orientation * Set Size * LSAS	0.762	0.387
Head Orientation * Set Size * AAX	1.839	0.182
Head Orientation * Eye Gaze	**21.138**	**<0.001**
Head Orientation * Eye Gaze * STAI-T	0.049	0.826
Head Orientation * Eye Gaze * LSAS	0.368	0.547
Head Orientation * Eye Gaze * AAX	0.038	0.846
Set Size * Eye Gaze	1.099	0.3
Set Size * Eye Gaze * STAI-T	1.365	0.249
Set Size * Eye Gaze * LSAS	0.159	0.692
Set Size * Eye Gaze * AAX	**6.288**	**0.016**
Head Orientation * Set Size * Eye Gaze	0.005	0.943
Head Orientation * Set Size * Eye Gaze * STAI-T	0.057	0.813
Head Orientation * Set Size * Eye Gaze * LSAS	1.793	0.187
Head Orientation * Set Size * Eye Gaze * AAX	0.207	0.652
**% Error Rates**		
**Set Size**	1.155	0.288
Set Size * STAI-T	0.431	0.515
Set Size * LSAS	0.01	0.921
Set Size * AAX	0.095	0.76
**Head Orientation**	**198.743**	**<0.001**
Head Orientation * STAI-T	0.136	0.714
Head Orientation * LSAS	0.519	0.475
Head Orientation * AAX	0.369	0.546
**Eye Gaze**	**12.037**	**0.001**
Eye Gaze * STAI-T	0.048	0.828
Eye Gaze * LSAS	1.339	0.253
Eye Gaze * AAX	0.026	0.871
Head Orientation * Set Size	0.013	0.908
Head Orientation * Set Size * STAI-T	0.408	0.526
Head Orientation * Set Size * LSAS	1.424	0.239
Head Orientation * Set Size * AAX	0.391	0.535
Head Orientation * Eye Gaze	37.158	**<0.001**
Head Orientation * Eye Gaze * STAI-T	0.158	0.693
Head Orientation * Eye Gaze * LSAS	1.139	0.291
Head Orientation * Eye Gaze * AAX	3.734	0.06
Set Size * Eye Gaze	1.716	0.197
Set Size * Eye Gaze * STAI-T	1.831	0.183
Set Size * Eye Gaze * LSAS	**5.303**	**0.026**
Set Size * Eye Gaze * AAX	0.895	0.349
Head Orientation * Set Size * Eye Gaze	14.828	**<0.001**
Head Orientation * Set Size * Eye Gaze * STAI-T	0.14	0.71
Head Orientation * Set Size * Eye Gaze * LSAS	**4.661**	**0.036**
Head Orientation * Set Size * Eye Gaze * AAX	**6.111**	**0.017**

## Results

### Questionnaire raw data

Table 1 summarizes the mean scores of our population for the STAI-T, LSAS, and AAX (from the RSQ), and reports their interrelations. There was a trend towards a positive relation between the STAI-T and LSAS scales.

### Response times and % error rates raw data

The means and standard deviations of the raw data acquired for response times and % error rates for each experimental condition are summarized in the Supplement (Section S1: Table S1 and Fig. S1).

### Response times and % error rates without personality

By concentrating on response times and % error rates during the target present condition, we then computed two 2 × 2 × 2 ANOVAs with the factors head orientation, set size, and eye gaze – one ANOVA for response times and one ANOVA for % error rates, respectively.

For response times, as expected, there was a significant main effect of set size (8 items > 4 items). Furthermore, there were significant main effects of head orientation (deviated > frontal) and eye gaze (averted > straight), and a significant interaction between head orientation and eye gaze. The latter interaction arose because response times were only significantly slower for averted as compared to straight gaze for the deviated head orientation: mean difference = 0.12, p < 0.001, 95% CI 0.082 to 0.161. Finally, we examined the presence of a search asymmetry effect by testing interactions with the additional factor set size. None of these interactions, however, were significant (ps > 0.33). Findings are depicted in Fig. 2.

For % error rates, there again was a significant main effect of set size (8 items > 4 items). In addition, there was a significant main effect of eye gaze (averted > straight), but no main effect of head orientation. Furthermore, there was a significant head orientation by eye gaze interaction. The latter interaction arose because % error rates were only significantly larger for averted as compared to straight gaze for the deviated head orientation: mean difference = 6.81, p < 0.001, 95% CI 4.97 to 8.64. We also examined search asymmetry, and here, these analyses showed a significant interaction between head orientation by eye gaze by set size. This effect was driven by the fact that the head orientation by eye gaze interaction was larger for set size 8 as compared to set size 4 (findings are depicted in Fig. 2). To delineate the underlying patterns within this interaction with even more detail, further decomposition steps were performed. These analyses as well as their results can be found in the Supplement (Section S2).

### Response times and % error rates with personality

After having assessed response times and % error rates as a function of head orientation, eye gaze, and set size across all participants irrespective of personality (see section above), we were interested in seeing whether participant personality (trait anxiety, social anxiety, and/or attachment anxiety) had any influence on the “stare-in-the-crowd” effect, both regarding search efficiency and search asymmetry. According to above analyses, we re-computed the 2 × 2 × 2 ANOVAs for response times and % error rates by adding all three questionnaire scales (STAI-T, LSAS, AAX) as covariates, and first concentrated on response times, followed by % error rates. In accordance with previous analyses showing significant two-way interactions between head orientation by eye gaze for both response rates and % error rates, as well as a significant three-way interaction between head orientation by eye gaze by set size for % error rates, we were mainly interested in assessing whether the anxiety personality scales showed any association with the above two-way or three-way interactions.

For response times, we did not observe any significant associations with personality regarding search efficiency. For search asymmetry, two significant interactions emerged, but not for the head orientation by eye gaze and/or by set size interaction. These interactions were therefore not followed up any further. Findings are summarized in Table 2.

**Figure 3:**
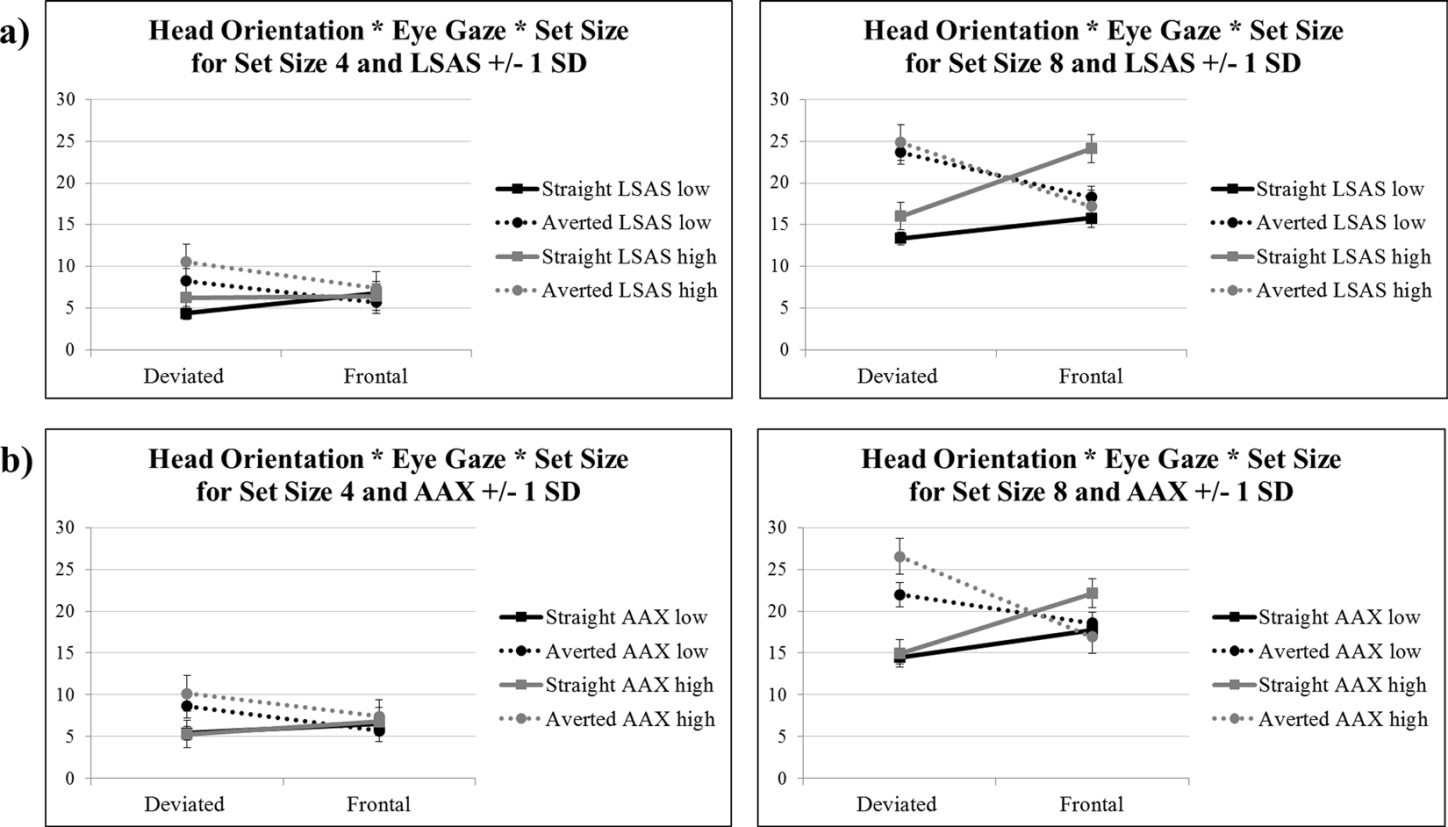
Illustration of the association between % error rate findings (y-axis) from the 2 × 2 × 2 ANOVA for the target present condition and social anxiety (LSAS) depicted in Panel (a), and attachment anxiety (AAX) depicted in Panel (b). Black lines represent personality values at -1 standard deviation (SD) below the mean (“low”), whereas grey lines represent personality values at +1 SD above the mean (“high”). Error bars represent 1 S.E.M. Depicted are estimated means from respective the 2 × 2 × 2 ANOVA.

Concerning % error rates, there were also no significant associations between search efficiency and personality. In turn, two significant relations emerged for search asymmetry in association with LSAS and AAX (see Table 2), and because these two significant interactions contained the full interaction between the factors head orientation by eye gaze by set size, they were followed up to further investigate their underlying pattern. To do so, estimates from the 2 × 2 × 2 ANOVA with covariates were extracted at LSAS and AAX scores +/− 1 standard deviation from the mean, respectively, and plotted. This procedure revealed that for participants scoring low on LSAS and/or AAX, there was a search asymmetry effect in terms of % error rates strongly resembling the search asymmetry pattern for the entire population (see above). For participants scoring high on LSAS and/or AAX, however, the search asymmetry effect differed. Participants scoring high on LSAS and/or AAX not only committed more errors for averted versus straight eye gaze on a deviated head targets, but also for straight versus averted eye gaze on a frontal head targets (see Fig. 3).

The estimates from the 2 × 2 × 2 ANOVAs including questionnaire scores to calculate the data illustrated in Fig. 3 are provided in the Supplement (Section S3: Table S2).

## Discussion

The present study investigated the “stare-in-the-crowd” effect by manipulating the experimental factors head orientation, set size, and eye gaze (with an additional target present vs. absent condition). In so doing, the aim was to (i) reproduce a head orientation by eye gaze interaction in response times and % error rates reflecting a search efficiency effect reported previously^24^, (ii) assess the presence of a search asymmetry effect not described so far, and (iii) determine whether inter-individual differences in three different kinds of anxiety were associated with distinct search efficiency and/or asymmetry effects. Analyses revealed a significant head orientation by eye gaze interaction (across set sizes 4 and 8, and irrespective of personality) in both response times and % error rates, and thus successfully replicated a previously reported search efficiency effect using a similar experimental paradigm. Furthermore, a search asymmetry effect was observed in % error rates (again across set sizes 4 and 8, and irrespective of personality), demonstrating that search asymmetry can also be observed when adding the factor head orientation to the original design. Regarding inter-individual differences in anxiety, several significant associations emerged regarding search asymmetry for response times and % error rates, of which two comprised an interaction between head orientation by eye gaze by set size regarding % error rates as a function of social anxiety and attachment anxiety and were thus followed up.

### Overall reproduction of eye gaze by head orientation interaction in search efficiency

As initially reported^24^, our data revealed that during the target present condition, search efficiency was comparable for targets with a frontal head orientation regardless of eye gaze direction, but selectively lower for targets with a deviated head orientation and an averted (versus straight) eye gaze. The same effect was also present for % error rates, with averted eye gaze on a deviated head targets selectively producing the highest amount of errors – a finding again according with Conty *et al*.^24^. Previous interpretation of such effect was based on the notion that combination of different eye gaze directions and head orientations may trigger distinct social cognitive processes in terms of social and/or behavioral intent, and that the averted eye gaze on a deviated head targets likely contained the least social and/or behavioral intent towards the observer. Therefore, slowest and worst detection of averted eye gaze on a deviated head targets was explained by the weakest attention-grabbing properties of this stimulus category^11,24^.

### Overall eye gaze by head orientation interaction in search asymmetry

By only manipulating the eye gaze direction during a visual search task investigating the “stare-in-the-crowd” effect, Cooper *et al*.^23^ describe a search efficiency effect and a search asymmetry effect because “… the inclusion of additional distractor items had a smaller effect on search latency for the direct-gaze target than it did for the averted-gaze target. In other words, search for the direct-gaze target was more efficient than search for the averted-gaze target”^23^. Because no search asymmetry effect has yet been found for the visual search task used to measure the “stare-in-the-crowd” effect including the factor head orientation, we also examined response times and % error rates for the presence of such search asymmetry effect in the current experiment. Our data revealed a significant search asymmetry effect for % error rates but not response times. While response times showed a comparable relative detection speed difference for averted versus straight eye gaze on a frontal head for set sizes 4 and 8, the % error rate difference for the same two experimental conditions on a deviated head significantly increased for set size 8 (as compared to set size 4). The above pattern suggests that participants did invest the same relative amount of time to search for targets with an averted (versus straight) eye gaze on a deviated head regardless of the amount of distractors present, which lead to relatively more committed errors for set size 8 (as compared to set size 4). Such failure to adjust response times to the increasing task demand to accurately detect targets with an averted (versus straight) eye gaze on a deviated head may once more be explained by the least social and/or behavioral intent towards the observer of this stimulus category^11,24^ (see above). In sum, using the eye region as stimuli, increasing the task demand of the visual search task seems critical in the emergence of the “stare-in-the-crowd” effect.

### Influence of inter-individual differences in anxiety on processing of eye gaze and head orientation

Besides looking for search efficiency and asymmetry effects across the entire sample of participants, we were also interested in determining whether inter-individual differences in trait, social, and/or attachment anxiety had an influence of the “stare-in-the-crowd” effect. We did not observe any effects of personality on search efficiency, neither in terms of response times, nor % error rates. However, several effects emerged when looking at search asymmetry, which reflects an increase in response times and % error rates for increasing task difficulty. The most specific and thus relevant finding was observed for social anxiety in terms of scores on the Leibowitz Social Anxiety Scale (LSAS) and attachment anxiety (AAX) questionnaires. For both the LSAS and AAX, we observed a differential eye gaze by head orientation by set size interaction as a function of increasing questionnaire scores. The underlying patterns, however, were not identical.

In case of LSAS, the association with the eye gaze by head orientation by set size interaction was driven by a selective increase in % error rates for the straight-gaze frontal head target condition. A possible explanation for this effect may be the presence of a particular strategy to avoid (prolonged) exposure to straight eye gaze on a frontal head by participants scoring high on social anxiety. Social anxiety is characterized by an intense fear of evaluation from others in social situations^50^. It has been linked to fear and avoidance of eye-to-eye contact (e.g.^51–54^) as a possible means to preclude the exposure to social evaluation that is perceived as a threat^55–59^. To successfully answer the question whether a given target is present or absent for set size 4, relatively little time focusing on each single item is required so that an extended exposure to the straight eye gaze on a frontal head target is not necessary. For set size 8, however, single items need to be inspected more closely and relatively more time needs to be spent on each item, including the straight eye gaze on a frontal head target. It may therefore be that a high LSAS score is reflected in less additional time spent on searching for a straight eye gaze on a frontal head target to avoid extended exposure to this condition – because it most closely resembles social evaluation –, which may entail an increase in the % error rate for target detection.

In case of AAX, the relation with the eye gaze by head orientation by set size interaction was not specifically driven by a single experimental condition, but rather a combination of decreased % errors committed for the averted-gaze frontal head and increased % errors committed for the straight-gaze frontal head condition. AAX is associated with the secondary attachment strategy of chronic hyperactivation of the attachment system, which is mainly linked to an increased sensitivity to negative social clues that could signal social rejection or abandonment^60^. Interestingly, the effects observed for AAX mainly concerned targets with a frontal head orientation, likely signaling stronger social intent than targets with a deviated head orientation. Furthermore, a decreased % error rate for averted-gaze frontal head targets implies a stronger attention capture of this condition, whereas an increased % error rate for straight-gaze frontal head targets suggests weaker attention capture of this condition. We may thus speculate that high anxiously attached participants’ attention was more strongly drawn to the averted-gaze frontal head condition because it possibly contained the strongest social clue of rejection or abandonment. Accordingly, when the number of distractors increased, these participants committed less errors in detecting averted-gaze frontal head targets within the same search time.

The above interpretations pertaining to social (LSAS) and attachment (AAX) anxiety in relation with the search asymmetry effect detected by increased % error rates (in the absence of such effect for response times) remain speculative and need replication and further extension. What nonetheless appears interesting is the fact that no specific effects in terms of a head orientation by eye gaze and/or by set size interaction were observed for trait anxiety (STAI-T) – although a more general effect was present for response times by means of a head orientation by set size by STAI-T interaction. We may therefore deliberate that this more general kind of anxiety was not associated with changes in response times and/or % error rates of one particular experimental condition because it did not change the processing of the conditions’ specific social meaning or significance conveyed by a combination of head orientation and eye gaze, and/or the latter processing under increased task demand.

### Constraints on Generality

The “stare-in-the-crowd” effect we report here in terms of search efficiency is a direct replication of a previous study^24^, and accords well with the overall literature on the “stare-in-the-crowd” effect for both search efficiency and asymmetry^23^. We nonetheless reckon that the present “stare-in-the-crowd” effect characterized by an eye gaze by head orientation interaction only represents the first independent replication since its original description in 2006, and that both the present study and the original investigation by Conty *et al*. were performed in a population of young adults. It therefore appears important to further replicate the observed “stare-in-the-crowd” effect in more varying participant populations.

Furthermore, it should be mentioned here that we used a specific set of stimuli only containing the eye region of a face, in accordance with the original description of the “stare-in-the-crowd” effect^22^ and the study the present investigation was based on^24^. However, the “stare-in-the-crowd” effect was shown to also be present when stimuli comprised the entire face of a person (see e.g.^23,25,26^), which points to (at least certain) generalizability across different facial stimuli. The above said, we do not have any evidence that these findings will occur outside of laboratory settings, which calls for future investigations of the “stare-in-the-crowd” effect in more naturalistic contexts.

In addition, we assessed inter-individual differences in trait, social, and attachment anxiety through self-report questionnaires in a healthy population. It therefore remains to be seen whether the observed findings also hold for clinical populations with a confirmed diagnosis (using other assessments than self-report questionnaires) of general or social anxiety disorder, as well as psychopathologies associated with attachment dysregulation such as borderline personality disorder (BPD) in adults, or attachment disorders in children.

In sum, we have no reason to believe that the results derived from the present investigation depend on characteristics of the participants, materials, or context, other than specified.

Our findings presented here replicate and importantly extend the available literature on the “stare-in-the-crowd” effect probing the factors eye gaze and head orientation. In addition, we found that the search asymmetry effect for % error rates was modulated by social and attachment but not trait anxiety. We may only speculate on the underlying mechanisms leading to such interaction pattern change, but nonetheless think that they may relate to the fear and avoidance of social evaluation characteristic for social anxiety, and hyperactivating secondary attachment strategies aiming at capturing clues for social rejection or abandonment characterizing attachment anxiety. These patterns warrant further experiments on the visual processing of social information in association with eye gaze direction and head orientation, especially regarding inter-individual differences in various kinds of social versus nonsocial anxiety.

## Supplementary information

Supplementary Data
