# Supplementary material for: Differential impact of trait, social, and attachment anxiety on the stare-in-the-crowd effect
Source: Sci Rep. 2019 Feb 11;9:1797. doi: 10.1038/s41598-019-39342-8 (PMC6370884; doi:10.1038/s41598-019-39342-8)
Supplement: Supplementary file 1 — Supplementary Data [file 41598_2019_39342_MOESM1_ESM.docx]

**Differential impact of trait, social, and attachment anxiety**

**on the stare-in-the-crowd effect**

Nicolas Burra^1*^, Solene Massait^1^, and Pascal Vrtička^2^

^1^Faculté de Psychologie et des Sciences de l’Education, Université de Genève, Geneva, Switzerland

^2^Max Planck Institute for Human Cognitive and Brain Sciences, Research Group Social Stress and Family Health, Leipzig, Germany

**SUPPLEMENTARY MATERIALS**

**S1: Raw Data**

| Response Times | | | | | | | | | | | | |
| --- | --- | --- | --- | --- | --- | --- | --- | --- | --- | --- | --- | --- |
|  |  | Frontal Head | | | | |  | Deviated Head | | | | |
|  |  | Target Present | |  | Target Absent | |  | Target Present | |  | Target Absent | |
| Array Size |  | Straight | Averted |  | Straight | Averted |  | Straight | Averted |  | Straight | Averted |
| 4 | *M* | 1096 | 1127 |  | 1243 | 1310 |  | 1138 | 1309 |  | 1298 | 1489 |
|  | *SD* | 197 | 249 |  | 226 | 303 |  | 203 | 266 |  | 199 | 298 |
| 8 | *M* | 1406 | 1417 |  | 1843 | 1772 |  | 1458 | 1641 |  | 1916 | 2147 |
|  | *SD* | 273 | 276 |  | 337 | 384 |  | 263 | 328 |  | 371 | 437 |
|  |  |  |  |  |  |  |  |  |  |  |  |  |
|  |  |  |  |  |  |  |  |  |  |  |  |  |
| % Error Rates | | | | | | | | | | | | |
|  |  | Frontal Head | | | | |  | Deviated Head | | | | |
|  |  | Target Present | |  | Target Absent | |  | Target Present | |  | Target Absent | |
| Array Size |  | Straight | Averted |  | Straight | Averted |  | Straight | Averted |  | Straight | Averted |
| 4 | *M* | 6.98 | 6.82 |  | 1.35 | 5.18 |  | 5.39 | 9.47 |  | 1.35 | 4.61 |
|  | *SD* | 5.79 | 7.52 |  | 2.19 | 12.75 |  | 3.77 | 6.85 |  | 2.02 | 7.85 |
| 8 | *M* | 19.96 | 17.78 |  | 1.65 | 5.92 |  | 14.61 | 24.45 |  | 1.84 | 5.39 |
|  | *SD* | 9.32 | 9.22 |  | 2.50 | 13.50 |  | 8.27 | 10.51 |  | 3.04 | 8.51 |

**Supplementary Table S1.** Mean raw response times (in milliseconds) and % error rates with standard deviation (SD) for N= 51 participants for all experimental conditions (also including target absent trials).


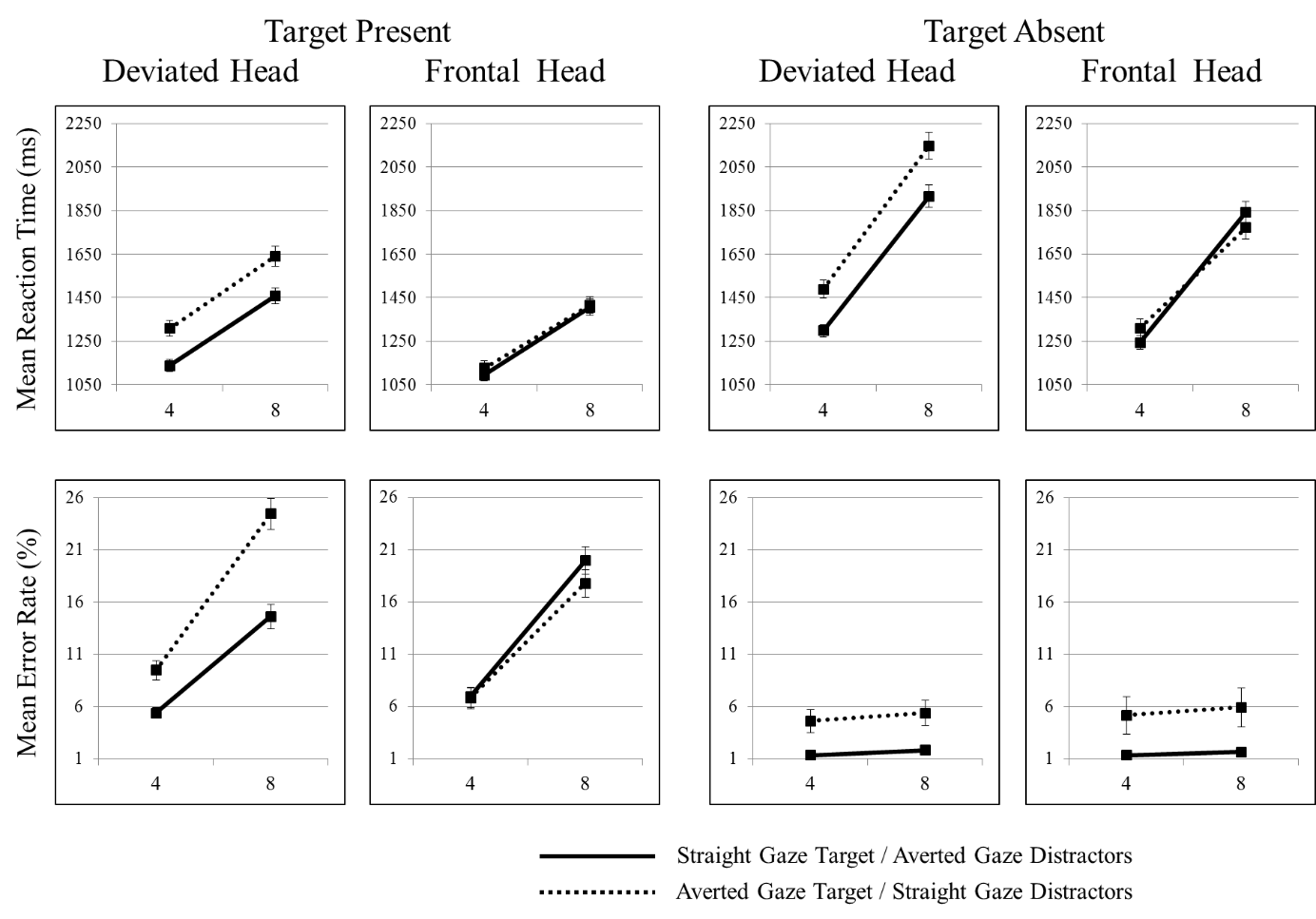


**Supplementary Figure S1.** Illustration of raw response times and % error rates. Top: mean response times (in milliseconds), and bottom: mean % error rates, for each of the experimental conditions (also including target absent trials). Error bars represent 1 S.E.M.

**S2: Decomposition of 2 x 2 x 2 ANOVA for % Error Rates**

The increase in % error rates from set size 4 to 8 for each of the four experimental conditions was estimated with an additional 2 x 2 repeated measures ANOVA with set size 8 minus 4 difference scores as dependent variables, revealing the following order: (1) averted eye gaze on deviated head: mean difference= 14.86, p< .001, 95% CI 11.65 to 18.07; (2) straight eye gaze on frontal head: mean difference= 13.00, p< .001, 95% CI 10.01 to 15.91; (3) averted eye gaze on frontal head: mean difference= 11.30, p< .001, 95% CI 8.87 to 13.72; and (4) straight eye gaze on deviated head: mean difference= 9.33, p< .001, 95% CI 7.2 to 11.47. Post-hoc comparisons of interest showed that the % error rates for the deviated head condition increased significantly stronger for averted as compared to straight eye gaze targets: mean difference= 5.53, p= .001, 95% CI 2.25 to 8.81, while for the frontal head condition, no differential increase as a function of target eye gaze was observed: mean difference= 1.71, p= .36, 95% CI -2.00 to 5.42. See Table 2 and Figure 2 for a summary and visual illustration.

**S3: Estimates of the Head Orientation by Eye Gaze by Set Size by LSAS / AAX Interaction used to Calclate Data illustrated in Figure 3**

| Estimates of the Head Orientation by Eye Gaze by Set Size by LSAS Interaction | | | | | | |
| --- | --- | --- | --- | --- | --- | --- |
| *LSAS -1 SD below Mean* | |  |  |  |  |  |
|  |  |  |  |  | *95% Confidence Interval* | |
| *Eye Gaze* | *Head Orientation* | *Set Size* | *Mean* | *STE* | *Low* | *High* |
| Straight | Deviated | 4 | 4.394 | 0.775 | 2.832 | 5.955 |
| Straight | Frontal | 8 | 13.362 | 1.658 | 10.022 | 16.702 |
| Straight | Deviated | 4 | 6.814 | 1.106 | 4.587 | 9.042 |
| Straight | Frontal | 8 | 15.775 | 1.704 | 12.343 | 19.206 |
| Averted | Deviated | 4 | 8.274 | 1.444 | 5.365 | 11.182 |
| Averted | Frontal | 8 | 23.676 | 2.124 | 19.398 | 27.954 |
| Averted | Deviated | 4 | 5.715 | 1.32 | 3.056 | 8.374 |
| Averted | Frontal | 8 | 18.285 | 1.969 | 14.32 | 22.251 |
|  |  |  |  |  |  |  |
|  |  |  |  |  |  |  |
| Estimates of the Head Orientation by Eye Gaze by Set Size by LSAS Interaction | | | | | | |
| *LSAS +1 SD above Mean* | |  |  |  |  |  |
|  |  |  |  |  | *95% Confidence Interval* | |
| *Eye Gaze* | *Head Orientation* | *Set Size* | *Mean* | *STE* | *Low* | *High* |
| Straight | Deviated | 4 | 6.293 | 0.834 | 4.613 | 7.973 |
| Straight | Frontal | 8 | 16.037 | 1.785 | 12.442 | 19.631 |
| Straight | Deviated | 4 | 6.47 | 1.19 | 4.072 | 8.867 |
| Straight | Frontal | 8 | 24.118 | 1.834 | 20.425 | 27.811 |
| Averted | Deviated | 4 | 10.543 | 1.554 | 7.413 | 13.674 |
| Averted | Frontal | 8 | 24.858 | 2.286 | 20.254 | 29.462 |
| Averted | Deviated | 4 | 7.415 | 1.421 | 4.553 | 10.277 |
| Averted | Frontal | 8 | 17.201 | 2.119 | 12.934 | 21.469 |
|  |  |  |  |  |  |  |
|  |  |  |  |  |  |  |
| Estimates of the Head Orientation by Eye Gaze by Set Size by AAX Interaction | | | | | | |
| *AAX -1 SD below Mean* | |  |  |  |  |  |
|  |  |  |  |  | *95% Confidence Interval* | |
| *Eye Gaze* | *Head Orientation* | *Set Size* | *Mean* | *STE* | *Low* | *High* |
| Straight | Deviated | 4 | 5.392 | 0.756 | 3.87 | 6.914 |
| Straight | Frontal | 8 | 14.47 | 1.617 | 11.214 | 17.726 |
| Straight | Deviated | 4 | 6.49 | 1.078 | 4.318 | 8.661 |
| Straight | Frontal | 8 | 17.76 | 1.661 | 14.415 | 21.105 |
| Averted | Deviated | 4 | 8.663 | 1.408 | 5.827 | 11.498 |
| Averted | Frontal | 8 | 21.991 | 2.071 | 17.821 | 26.161 |
| Averted | Deviated | 4 | 5.693 | 1.287 | 3.101 | 8.285 |
| Averted | Frontal | 8 | 18.575 | 1.919 | 14.71 | 22.441 |
|  |  |  |  |  |  |  |
|  |  |  |  |  |  |  |
| Estimates of the Head Orientation by Eye Gaze by Set Size by AAX Interaction | | | | | | |
| *AAX +1 SD above Mean* | |  |  |  |  |  |
|  |  |  |  |  | *95% Confidence Interval* | |
| *Eye Gaze* | *Head Orientation* | *Set Size* | *Mean* | *STE* | *Low* | *High* |
| Straight | Deviated | 4 | 5.295 | 0.773 | 3.737 | 6.852 |
| Straight | Frontal | 8 | 14.928 | 1.654 | 11.597 | 18.26 |
| Straight | Deviated | 4 | 6.794 | 1.103 | 4.572 | 9.016 |
| Straight | Frontal | 8 | 22.133 | 1.699 | 18.71 | 25.555 |
| Averted | Deviated | 4 | 10.155 | 1.44 | 7.254 | 13.056 |
| Averted | Frontal | 8 | 26.543 | 2.119 | 22.276 | 30.81 |
| Averted | Deviated | 4 | 7.437 | 1.317 | 4.784 | 10.089 |
| Averted | Frontal | 8 | 16.912 | 1.964 | 12.957 | 20.867 |

**Supplementary Table S2**. Summary of estimates used to calculate data illustrated in Figure 3 from the 2 x 2 x 2 ANOVAs comprising questionnaire scores. SD= standard deviation, STE= standard error

**S4: 2 x 2 x 2 ANOVAs for Reaction Times and % Error Rates and Personality for each Covariate (STAI-T, LSAS, AAX) separately**

Below, we report the full 2 x 2 x 2 ANOVAs for reaction times and % error rates with trait anxiety (STAI-T), social anxiety (LSAS), and attachment anxiety (AAX) as covariates (by controlling for age and sex) added separately (i.e. 3 ANOVAs for reaction times and 3 ANOVAs for % error rates).

| RESPONSE TIMES with STAI-T only |  |  |
| --- | --- | --- |
| *Factor* | *F-Value* | *p-Value* |
| Set Size | **42.141** | **< .001** |
| Set Size * STAI-T | 0.142 | 0.708 |
| Head Orientation | **640.628** | **< .001** |
| Head Orientation * STAI-T | 0.69 | 0.41 |
| Eye Gaze | **30.875** | **< .001** |
| Eye Gaze * STAI-T | 0.506 | 0.48 |
| Head Orientation * Set Size | 0.006 | 0.94 |
| Head Orientation * STAI-T | **6.531** | **0.014** |
| Head Orientation * Eye Gaze | **21.614** | **< .001** |
| Head Orientation * STAI-T | 0.131 | 0.719 |
| Set Size * Eye Gaze | 0.974 | 0.329 |
| Set Size * STAI-T | 2.033 | 0.16 |
| Head Orientation * Set Size * Eye Gaze | 0.001 | 0.973 |
| Head Orientation * Set Size * Eye Gaze * STAI-T | 0.025 | 0.874 |
|  |  |  |
| RESPONSE TIMES with LSAS only |  |  |
| *Factor* | *F-Value* | *p-Value* |
| Set Size | **42.681** | **< .001** |
| Set Size * LSAS | 2.677 | 0.108 |
| Head Orientation | **631.376** | **< .001** |
| Head Orientation * LSAS | 0.361 | 0.551 |
| Eye Gaze | **30.638** | **< .001** |
| Eye Gaze * LSAS | 0.044 | 0.835 |
| Head Orientation * Set Size | 0.036 | 0.85 |
| Head Orientation * LSAS | 2.201 | 0.145 |
| Head Orientation * Eye Gaze | **22.152** | **< .001** |
| Head Orientation * LSAS | 0.493 | 0.486 |
| Set Size * Eye Gaze | 0.925 | 0.341 |
| Set Size * LSAS | 0.025 | 0.875 |
| Head Orientation * Set Size * Eye Gaze | 0.003 | 0.953 |
| Head Orientation * Set Size * Eye Gaze * LSAS | 1.791 | 0.187 |
|  |  |  |
| RESPONSE TIMES with AAX only |  |  |
| *Factor* | *F-Value* | *p-Value* |
| Set Size | **43.105** | **< .001** |
| Set Size * AAX | 1.591 | 0.213 |
| Head Orientation | **638.427** | **< .001** |
| Head Orientation * AAX | 0.569 | 0.454 |
| Eye Gaze | **30.574** | **< .001** |
| Eye Gaze * AAX | 0.042 | 0.838 |
| Head Orientation * Set Size | 0.005 | 0.944 |
| Head Orientation * AAX | 0.909 | 0.345 |
| Head Orientation * Eye Gaze | **21.555** | **< .001** |
| Head Orientation * AAX | 0.032 | 0.858 |
| Set Size * Eye Gaze | 1.205 | 0.278 |
| Set Size * AAX | **7.548** | **0.008** |
| Head Orientation * Set Size * Eye Gaze | 0.001 | 0.979 |
| Head Orientation * Set Size * Eye Gaze * AAX | 0.155 | 0.695 |
|  |  |  |
| % ERROR RATES with STAI-T only |  |  |
| *Factor* | *F-Value* | *p-Value* |
| Set Size | 1.214 | 0.276 |
| Set Size * STAI-T | 0.461 | 0.501 |
| Head Orientation | **203.12** | **< .001** |
| Head Orientation * STAI-T | 0.445 | 0.508 |
| Eye Gaze | **12.846** | **0.001** |
| Eye Gaze * STAI-T | 0.251 | 0.619 |
| Head Orientation * Set Size | 0.002 | 0.968 |
| Head Orientation * STAI-T | 0.769 | 0.385 |
| Head Orientation * Eye Gaze | **34.083** | **< .001** |
| Head Orientation * STAI-T | 0.02 | 0.889 |
| Set Size * Eye Gaze | 2.073 | 0.157 |
| Set Size * STAI-T | **4.221** | **0.046** |
| Head Orientation * Set Size * Eye Gaze | **11.405** | **0.001** |
| Head Orientation * Set Size * Eye Gaze * STAI-T | 0.236 | 0.629 |
|  |  |  |
| % ERROR RATES with LSAS only |  |  |
| *Factor* | *F-Value* | *p-Value* |
| Set Size | 1.147 | 0.29 |
| Set Size * LSAS | 0.08 | 0.778 |
| Head Orientation | **205.03** | **< .001** |
| Head Orientation * LSAS | 0.703 | 0.406 |
| Eye Gaze | **12.507** | **0.001** |
| Eye Gaze * LSAS | 1.644 | 0.206 |
| Head Orientation * Set Size | 0.021 | 0.885 |
| Head Orientation * LSAS | 2.048 | 0.159 |
| Head Orientation * Eye Gaze | **35.323** | **< .001** |
| Head Orientation * LSAS | 0.885 | 0.352 |
| Set Size * Eye Gaze | 1.638 | 0.207 |
| Set Size * LSAS | **7.137** | **0.01** |
| Head Orientation * Set Size * Eye Gaze | **13.254** | **0.001** |
| Head Orientation * Set Size * Eye Gaze * LSAS | 3.98 | 0.052 |
|  |  |  |
| % ERROR RATES with AAX only |  |  |
| *Factor* | *F-Value* | *p-Value* |
| Set Size | 1.202 | 0.279 |
| Set Size * AAX | 0.047 | 0.83 |
| Head Orientation | **203.496** | **< .001** |
| Head Orientation * AAX | 0.441 | 0.51 |
| Eye Gaze | **12.779** | **0.001** |
| Eye Gaze * AAX | 0.025 | 0.875 |
| Head Orientation * Set Size | 0.001 | 0.971 |
| Head Orientation * AAX | 0.313 | 0.578 |
| Head Orientation * Eye Gaze | **37.055** | **< .001** |
| Head Orientation * AAX | 3.568 | 0.065 |
| Set Size * Eye Gaze | 1.862 | 0.179 |
| Set Size * AAX | 1.076 | 0.305 |
| Head Orientation * Set Size * Eye Gaze | **12.97** | **0.001** |
| Head Orientation * Set Size * Eye Gaze * AAX | **5.437** | **0.024** |

**Supplementary Table 3.** Results of the three 2 x 2 x 2 ANOVAs with the factors head orientation, eye gaze, and set size, including the covariates trait anxiety (STAI-T), social anxiety (LSAS), and attachment anxiety (AAX) separately, regarding response times (top) and % error rates (bottom). All significant interactions are highlighted in bold.
